# Characterization of Vaginal *Escherichia coli* Isolated from Pregnant Women in Two Different African Sites

**DOI:** 10.1371/journal.pone.0158695

**Published:** 2016-07-07

**Authors:** Emma Sáez-López, Anélsio Cossa, Rachid Benmessaoud, Lola Madrid, Cinta Moraleda, Sonia Villanueva, Houssain Tligui, Benilde Moiane, Hassan Alami, Sérgio Massora, Rachid Bezad, Inacio Mandomando, Jordi Bosch, Jordi Vila, Quique Bassat, Sara M. Soto

**Affiliations:** 1 Department of Clinical Microbiology, Hospital Clínic—Universitat de Barcelona, Barcelona, Spain; 2 ISGlobal, Barcelona Ctr. Int. Health Res. (CRESIB), Hospital Clínic—Universitat de Barcelona, Barcelona, Spain; 3 Centro de Investigação em Saúde de Manhiça (CISM), Maputo, Mozambique; 4 Équipe de Recherche en Santé et Nutrition du Couplé Mère Enfant, Faculté de Médecine et de Pharmacie, Université Mohammed V Souissi, Rabat, Morocco; 5 Équipe de Recherche de Périnatologie, Université Mohammed V Souissi, Rabat, Morocco; State University of Maringá/Universidade Estadual de Maringá, BRAZIL

## Abstract

The relevance of vaginal colonization of pregnant women by *Escherichia coli* is poorly understood, despite these strains sharing a similar virulence profile with other extraintestinal pathogenic *E*. *coli* producing severe obstetric and neonatal infections. We characterized the epidemiology, antimicrobial susceptibility and virulence profiles of 84 vaginal *E*. *coli* isolates from pregnant women from Rabat (Morocco) and Manhiça (Mozambique), two very distinct epidemiological settings. Low levels of antimicrobial resistance were observed to all drugs tested, except for trimethoprim-sulfamethoxazole in Manhiça, where this drug is extensively used as prophylaxis for opportunistic HIV infections. The most prevalent virulence factors were related to iron acquisition systems. Phylogroup A was the most common in Rabat, while phylogroups E and non-typeable were the most frequent in Manhiça. Regardless of the apparently “low virulence” of these isolates, the frequency of infections is higher and the outcomes more devastating in constrained-resources conditions, especially among pregnant women and newborns.

## Introduction

Most extraintestinal infections associated with *Escherichia coli* are caused by commensal strains that become pathogenic by adaptation stratagems or the acquisition of virulence determinants (Extraintestinal Pathogenic *E*. *coli* (ExPEC)) [1] and subsequently reach a particular sterile body site [2]. Although vaginal *E*. *coli* (VEC) share a virulence profile with ExPEC [3], the presence of *E*. *coli* in the vagina has been poorly characterized, in contrast to other anatomic sites. VEC isolates are able to cause several asymptomatic and symptomatic infections [4]. Furthermore, there is a clear link between *E*. *coli* forming part of the genital tract flora and those causing intra-amniotic infection [5], preterm premature rupture of membranes (PPROM) [6], preterm delivery <34 weeks and the consequent neonatal outcomes, including very-low birth weight newborns [7] and neonatal sepsis [3]. The prevalence of perinatal transmission of *E*. *coli* during delivery ranges between 21 to 50% [8], being a clear predisposing factor to develop neonatal infections [9]. *E*. *coli* is the most frequent Gram-negative bacteria involved in neonatal meningitis (45–64%) [10] and is the most common cause of stillbirth in developing countries [11].

Antibiotic resistance varies from one geographical region to another [12]. However, the high resistance of empiric antibiotherapy for different diseases is making increasingly difficult to provide effective antibiotic treatment in Africa [12–14]. In addition, the number of multidrug-resistant strains is increasing, being extended spectrum β-lactamases (ESBLs) and carbapenemases a matter of great concern [15–16].

Pertenance to B2 phylogenetic group and possession of virulence factors, such as adhesins, iron acquisition systems, invasins and toxins have been associated with ExPEC causing obstetric and perinatal complications [16–17]. Different distribution patterns have been described according to geographic conditions, habits and host-associated factors [18].

The frequency and the consequences of infections transmitted from mother-to-child differ according to the socio-economic conditions of each geographic area and are more common and devastating in low-income countries with high exposure to infectious organisms, nutrient deficiencies [9], low immune response [11], and limited access to health care services [10]. Therefore, it is important to characterize VEC isolates to determine their virulence potential and ability to cause obstetric and neonatal infections.

This study was undertaken to determine the epidemiology, antimicrobial susceptibility and virulence profiles of VEC isolates from pregnant women from two very different sites in Africa, namely Rabat (Morocco, Northern Africa) and Manhiça (Mozambique, Southern Africa). The rationale for recruiting in these two very distinct sites was to obtain a snapshot of the colonization of pathogens among pregnant women under two very distinct epidemiological conditions, not only in terms of circulating pathogens (with a very high HIV prevalence documented in Manhiça, [19] and virtually no HIV in Rabat; and malaria being endemic in Manhiça but not in Morocco [20]) but also in terms of antibiotic usage in those two settings [21].

## Materials and Methods

### Clinical sample collection and identification of *E*. *coli* isolates

The study included vaginal samples from pregnant women collected at the *Maternité des Orangers*, in Rabat from March to July 2013, and at the *Hospital de Manhiça*, in Manhiça from June 2014 to January 2015. Pregnant women were recruited within the framework of two projects: (i) “Determining the epidemiology and risk factors for *Group B Streptococcus* and *E*.*coli* and other bacterial infections among pregnant women and newborns in Rabat, Morocco (EFRIMN)” and (ii) “Perinatal Group B Streptococcus, *Escherichia coli* and *Pneumocystis jirovecii* infections in pregnant women and newborns in Manhiça, Mozambique (PIPAC)”. All participating women signed a written informed consent prior to their inclusion in the study. Protocols have been approved by the Ehical Committee of the Hospital Clinic of Barcelona, the Ethical committee of the “*Faculté de Médecine et Phamacie de l’Université Mohammed V-Souissi”* of Rabat and the Ethical Committee of the Centro de Investigação em Saúde de Manhiça (CISM). Samples were collected at two different times, either (A) during the antenatal control at ≥ 35 weeks of pregnancy and (B) or at delivery, regardless gestational age. All the samples were spread onto MacConkey agar and incubated at 37°C overnight. The isolates were identified based on colony appearance, Gram stain and standard biochemical tests. Some identifications were confirmed by MALDI-TOF.

### Antimicrobial resistance

Resistance profiles were determined using the standard Kirby-Bauer disk-diffusion method following CSLI guidelines [22]. The *E*. *coli* ATCC 25922 strain was used as the control. The antimicrobial agents tested were ampicillin (AMP: 10 μg), amoxicillin/clavulanic acid (AMC: 30 μg), cefuroxime (CXM: 30 μg), cefotaxime (CTX: 30 μg), ceftazidime (CAZ: 30 μg), imipenem (IPM: 10 μg), ertapenem (ETP: 10 μg), meropenem (MEM: 10 μg), tetracycline (TET: 30 μg), trimethoprim-sulfamethoxazole (SXT: 30μg), gentamicin (GEN: 10μg), nalidixic acid (NAL: 30 μg), ciprofloxacin (CIP: 5 μg), chloramphenicol (CHL: 30 μg), aztreonam (AZT: 15 μg), piperacillin/tazobactam (TZP: 100/10 μg), fosfomycin (FOF: 200 μg), and colistin (CST: 10 μg). In addition, ESBLs were identified by the double-disk synergy test using CTX, AMC and CAZ.

### Prevalence of virulence factor genes (VFGs)

Thirteen virulence factor genes (VFGs) associated with ExPEC were detected by polymerase chain reaction (PCR) using specific primers [23]. The genes studied included: hemolysin (*hly*A), cytotoxic necrotizing factor (*cnf*1), autotrasporter toxin (*sat*1), P- fimbriae (*pap*A, -EF, -C), type 1-C fimbriae (*foc*G), heat-resistant hemagglutinin (*hra*), yersiniabactin (*fyu*A), siderophores (*iut*A and *iro*N), aerobactin (*iuc*C) and invasion of brain endothelium factor (*ibe*A). PCR reactions were carried out under the following conditions: 94°C for 3 min, 25 cycles of denaturation at 94°C for 30 s, the corresponding annealing temperature (55–63°) for 30 s, 72°C for 1 min and 30 s, and a final elongation at 72°C for 7 min. Samples were run in 1.5% agarose gels and stained with Syber Safe. A 100 bp DNA ladder was used in each gel as a molecular size marker and positive and negative control strains for the traits were included.

### Phylogenetic analysis

The new *E*. *coli* phylotyping method proposed by Clermont et al. [24] was used to classify the strains into 8 groups (A, B1, B2, C, D, E, F, and *Escherichia* cryptic clade I). A few modifications were made, including the use of a quadruplex-PCR instead of a simplex PCR method to determine the *arp*A, *chu*A, *yja*A genes and the DNA fragment TspE4.C2. In addition, a primer concentration of 20 μM was used and a simple PCR was performed for the second screening to differentiate between group A or C and E or D, respectively.

### Statistical analysis

Statistical analyses were performed using IBM SPSS Statistics software for Windows, version 20.0. We compared antibiotic resistance, the presence of VFGs and the phylogenetic groups of all the isolates, considering a p-value below 0.05 as significant and p-values below 0.01 as statistically significant. Categorical data were compared using the Chi-squared test and Yates' correction for continuity was applied to avoid overestimation of statistical significance when the number of expected cases was between 3–5 in at least one group. When the number of expected cases was below 3 in at least one group, the Fisher’s exact test was used.

## Results

### Collection of bacterial isolates

A total of 33 VEC isolates (A = 18, B = 15) and 51 VEC isolates (A = 24, B = 27) were isolated from pregnant women in Rabat and in Manhiça, respectively.

### Antimicrobial resistance

**Fig 1:**
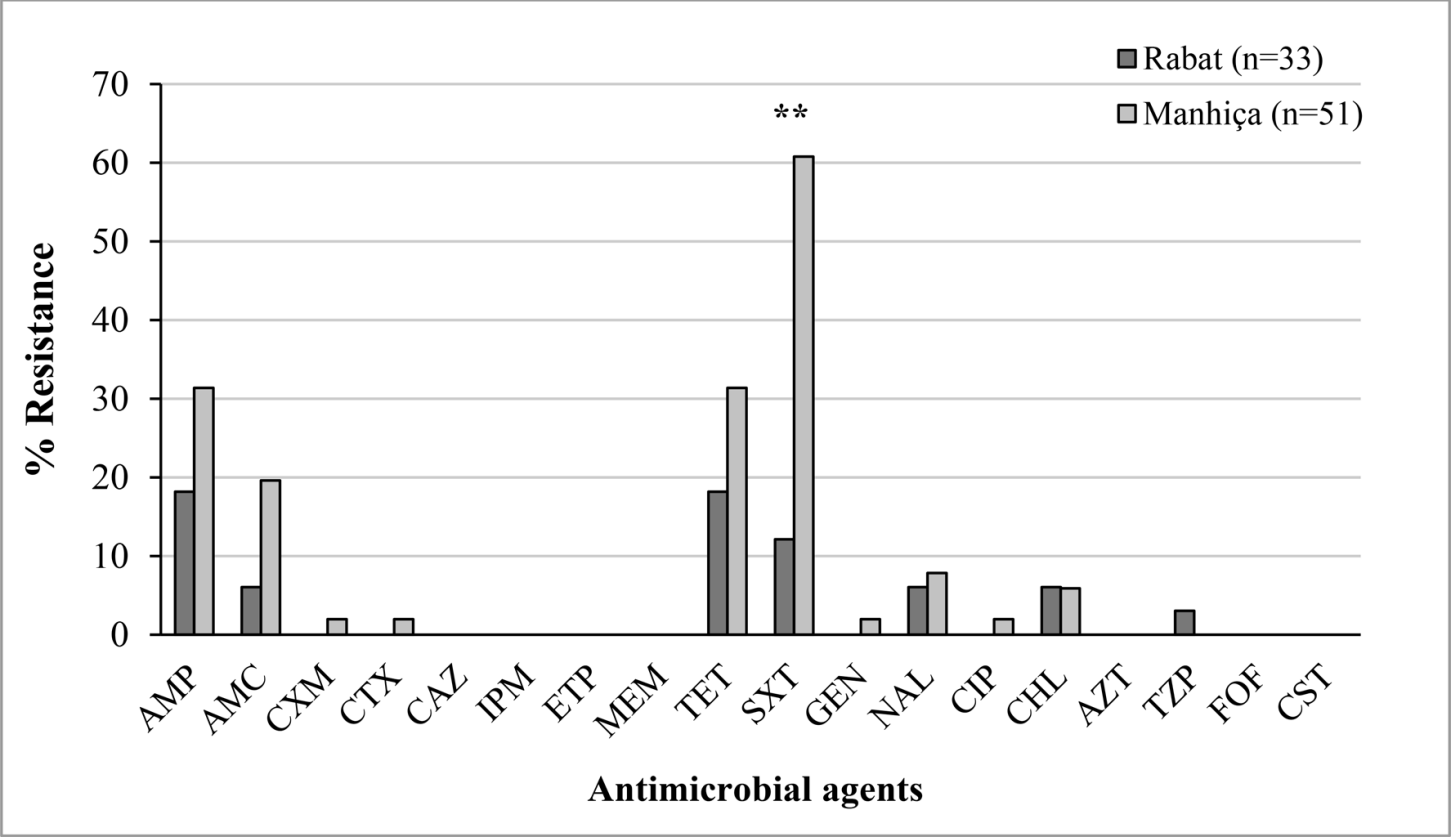
Distribution of antimicrobial resistance among *E*. *coli* isolates from Rabat and Manhiça. AMP, ampicillin; AMC, amoxicillin/clavulanic acid; CXM, cefuroxime, CTX, cefotaxime; CAZ, ceftazidime; IPM, imipenem; ETP, ertapenem; MEM, meropenem; TET, tetracycline, SXT, trimethoprim/ sulfamethoxazole; GEN, gentamicin; NAL, nalidixic acid; CIP, ciprofloxacin; CHL, chloramphenicol; AZM, azithromycin; TZP, piperacillin/tazobactam; FOF, fosfomycin, and CST, colistin. (**P-value < 0.0001).

All the isolates were screened for susceptibility to 17 antimicrobial agents **(Fig 1)**. We classified isolates showing intermediate levels of susceptibility as resistant. Susceptibility to all antimicrobial agents tested was seen in 22 isolates (67%) in Rabat and in 14 isolates (27%) in Manhiça, with this difference being statistically significant (P = 0.001). Only one ESBL-producing *E*. *coli* (harboring a CTX-M-15 enzyme) was found in one isolate from Manhiça. Frequency of resistance to each of the antimicrobial agents studied, with the exception of CHL, was higher in Manhiça compared to Rabat. The difference was statistically significantly only in the case of the resistance to SXT (61% vs. 12% in Manhiça and Rabat, respectively, P < 0.0001). No significant differences were found between *E*. *coli* isolates from women belonging to group A or B.

### Prevalence of VFGs

Remarkably, none of the virulence factors studied was encoded in 21% (7/33) of the strains from Rabat compared with 43% (22/51) of those from Manhiça. The isolates from Rabat presented a higher frequency of VFGs than those from Manhiça, except for *fyu*A and *ibe*A, showing statistical significance in the case of *iut*A (58 *vs*. 31%, P = 0.021), *iuc*C (55 *vs*. 27%, P = 0.024) and the hemolysin *hly*A (21% *vs*. 2%, P = 0.005) **(Table 1)**. In general, iron acquisition systems were the most frequent VFGs observed (*iuc*C, 42%; *fyu*A, 40%; *iut*A, 38%), while the *cnf*1 (2%), *foc*G (2%) and *ibe*A (5%) genes were the least frequent. No statistically significant differences were observed between groups A and B.

**Table 1:** Prevalence of virulence factor genes (VFGs) among *E*.*coli* isolates from Rabat and Manhiça.

	Geographic area		
VFGs^a^	Rabat/Morocco n = 51 (%)	Manhiça/Mozambique n = 33 (%)	P-value
*hly*A	**7 (21)**	**1 (2)**	0.005**
*cnf*1	2 (6)	0	0.151
*sat*1	10 (30)	10 (20)	0.301
*pap*A	5 (15)	5 (10)	0.504
*pap*EF	5 (15)	5 (10)	0.504
*pap*C	6 (18)	6 (12)	0.526
*foc*G	2 (6)	0	0.151
*hra*	10 (30)	7 (14)	0.095
*fyu*A	16 (48)	18 (35)	0.261
*iut*A	**18 (55)**	**14 (27)**	0.021*
*iro*N	5 (15)	4 (8)	0.306
*iuc*C	**19 (58)**	**16 (31)**	0.024*
*ibe*A	1 (3)	3 (6)	1

^a^VFGs: Virulence Factor Genes

*p-value<0.05

**p-value = 0.005

### Phylogenetic analysis

**Fig 2:**
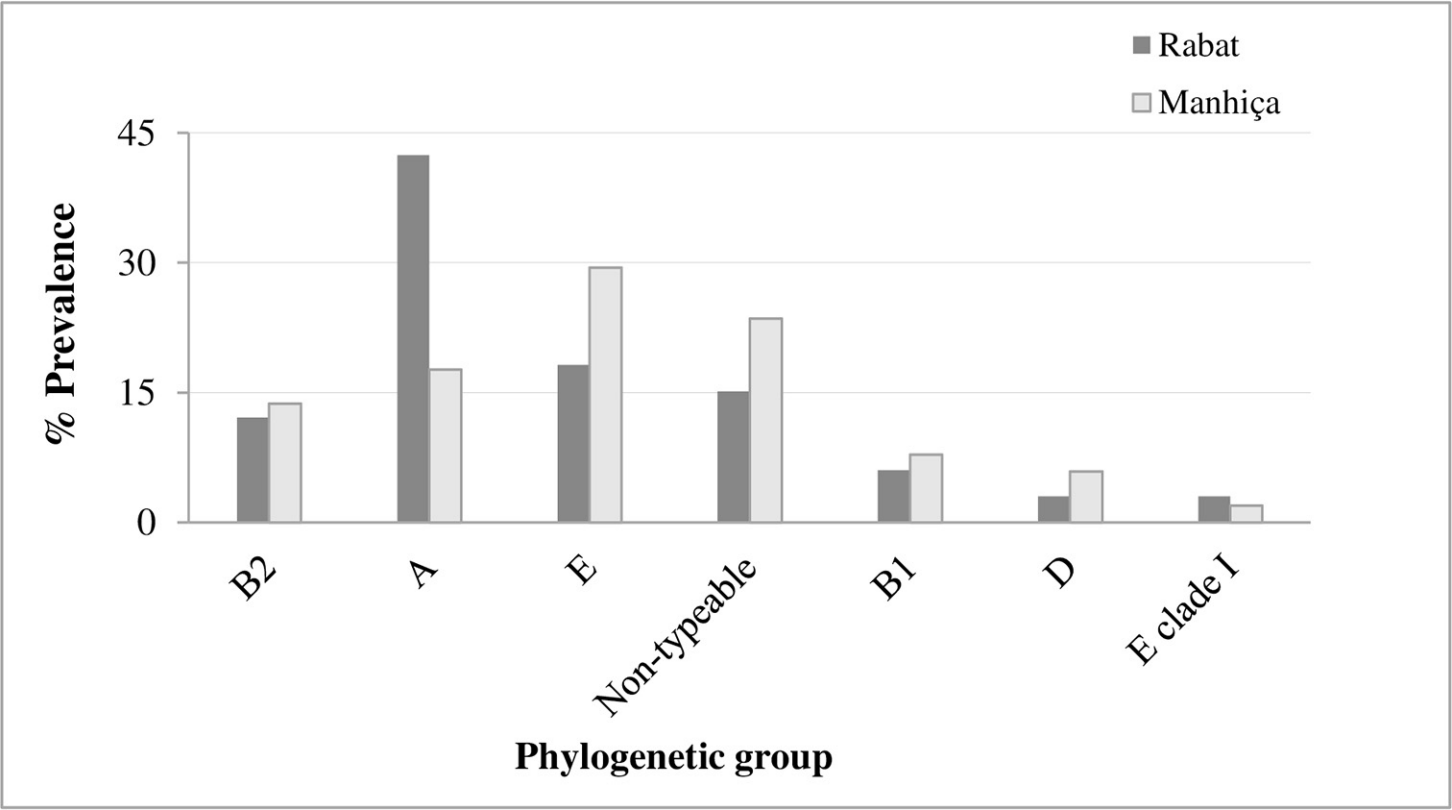
Distribution of phylogenetic groups among *E*.*coli* isolates from Rabat and Manhiça.

Phylogenetic group A was the most common group among all the isolates from Rabat (14/33, 42%), whereas group E and non-typeable isolates were most frequently isolated in Manhiça (15/51, 29% and 12/51, 23.5%, respectively) **(Fig 2)**. Four (12%) and seven (14%) strains from Rabat and Manhiça, respectively, belonged to phylogroup B2. Phylogroups B1, D and E clade I were identified in 12% of the remaining isolates from Rabat and phylogroups B1, D and F were identified in 16% of those from Manhiça. No differences were found between groups A and B in the two geographical areas.

### Relationship between virulence factors, phylogenetic group and antimicrobial resistance

No association was found between phylogroup B2 and VFGs. On the other hand, a statistically significant relationship was observed between SXT-resistance and the presence of the siderophores *fyu*A (P = 0.018) and *iut*A (P = 0.029) in Manhiça., Only in the case of ampicillin-resistant isolates, the frequency of phylogroups B2 and D was higher than statistically expected (18% *vs*. 13% and 18% *vs*. 5%, respectively).

## Discussion

Pregnant women are more prone to develop different types of extraintestinal *E*. *coli* infections, especially in the third trimester [14]. Host factors and the anatomic origin of the infection play an important role in the severity of the disease [25]. Vagina and endocervix are reservoirs for the colonization of *E*. *coli* strains and act favoring invasion of the amniotic fluid with the subsequent risk of neonatal infections [26]. There are few studies on the antimicrobial susceptibility and/or virulence of *E*. *coli* isolates colonizing the genital tract of pregnant women [3],[23],[26–27]. Moreover, to our knowledge no studies have been carried out to this respect in low or middle-income countries (LMICs), despite worse outcomes presented in these areas.

No differences in neither features studied were found between isolates from women attending antenatal clinics and those recruited during delivery, suggesting that sampling at delivery is equally effective in terms of detecting pathogens (and defining their virulence profiles). No changes seem to occur in terms of virulence profiles in infections acquired early or late during pregnancy. Treating infections in LMICs adequately is challenging, because of the increasing resistance to first line treatments. This is due to the extended use and misuse of antibiotics [14–15],[28], the prohibitive costs in such settings of alternative choices such as cephalosporins [29] and the limited antibiotic stock available in these areas [30]. Our isolates showed remarkably low antimicrobial resistance levels compared to ExPEC isolates from other studies carried out in several African countries [14–15]. Notably, two thirds of the Moroccan (Rabat) isolates from this series were completely susceptible. On the other hand, high resistance to SXT was found in Manhiça, similar to the results of a previous study on diarrheagenic *E*. *coli* [29] carried out in the same area. Indeed, resistance to this drug has been on the rise due to its low cost and extensive use as prophylaxis to prevent HIV-opportunistic infections and other community-acquired infections [31]. Surprisingly, in contrast to several reports which described an increase in the prevalence of strains harboring ESBLs, we only found one ESBL-producing isolate [14],[32]. We also detected fewer gentamicin and ampicillin-resistant strains than previous studies carried out in other countries [23], [27],[33–34], including the aforementioned study in the same center [29]. These results suggest that empirical treatment recommended by the World Health Organization for chorioamnionitis [35] and neonatal sepsis and/or meningitis (ampicillin plus an aminoglycoside, normally gentamicin) and postpartum endometritis [36] (clindamycin or ampicillin plus gentamicin) could be effective.

In general, the isolates studied could not be considered as very virulent because of the low prevalence of VFGs found compared to other studies [3],[23],[27],[37], particularly in regard to the 43% of our isolates from Manhiça which did not encode any VFG. However, the presence of the *hly*A gene, an indicator of virulence and the possible cause of intestinal and extraintestinal infections [37–38], the *iut*A gene, a siderophore, and the *iuc*C gene, an aerobactin, which plays a specific role in maternofetal infections [26], was significantly more frequent in VEC isolates from Rabat than in those from Manhiça, suggesting that these isolates would be major virulence determinants carriers. Similar to previous studies [27], iron transport systems were the virulence factors most frequently found in isolates from both sites. These systems could be targets for future vaccination strategies in urinary tract infections (UTIs) [39]. Indeed, the siderophore, *iut*A, and the yersiniabactin, *fyu*A, were significantly more frequently observed among SXT-resistant compared to the susceptible isolates in Manhiça, contrary to what was reported by Moreno et al. [40], indicating that in our case the selective pressure of SXT had not effects in the virulence carriage of these strains.

Less than 15% of isolates from the two countries belonged to the B2 phylogroup, in contrast to previous reports of more than 50% [23],[26–27]. The most predominant phylogroup in Manhiça was E. This phylogroup is known for the well-recognized O157:H7, and these strains are associated with the sharing of alleles with groups B1 and A [41], and the absence of virulence factors, bacteriocins and antimicrobial resistance [42]. One quarter of the isolates from Manhiça were non-typeable, since they could not be assigned to any phylogroup or indicated the high variability of gene content due to the gain or loss of virulence genes [24]. This result was similar to what described in a study analyzing uropathogenic *E*. *coli* isolates performed in Iran [43] but differs from the results obtained by Clermont et al. [24], who reported that phylogrouping was not possible in less than 1% of the isolates studied. This fact has been related to the variability found in phylogenetic group distribution according to several factors [18]. Phylogroup A was the most frequently detected in Rabat, being associated with a low number of VFGs and commensal flora. However, it is not clear whether *E*. *coli* isolates should be defined as commensal based entirely on the source and/or regarding the phylogenetic group since phylogroups A and B1 may cause extraintestinal infections in immunocompromised hosts at a specific time [44]. Taking all this information into account, the virulence of these VEC isolates was low according to the phylogenetic group and the possession of VFGs compared with other studies from East Japan [3], France [26], and Spain [23],[27], in which most of the strains studied belonged to group B2 and had a significant number of VFGs. However, in our study isolates belonging to phylogroups B2 and D were more frequent among ampicillin-resistant isolates while in other studies, non-B2 phylogroups were associated with resistant strains, albeit to other antimicrobial agents [40].

One limitation of the present study is that it is focused on colonization rather than infection and included only two hospitals from different sites of Africa, thereby not allowing extrapolation of our results to LMICs. However, this is the first study to characterize VEC isolates from pregnant women in these countries. Considering the involvement of VEC isolates in extraintestinal infections, our results may be helpful to develop strategies to prevent maternal and neonatal infections. Furthermore, vaginal swabs have shown to be a good tool to guide proper clinical management in women presenting PPROM in resource-limited sites where other more complex microbiological methods are not viable [6].

In summary, VEC isolates from Rabat and Manhiça showed low virulence and antimicrobial resistance compared to isolates from developed countries suggesting that they are not intrinsically ‘virulent clones’. However, the emergence of multidrug resistance, the plasticity of the genome of *E*. *coli* isolates which allows the acquisition of virulence genes, the relationship between disease severity and host factors, the vulnerability to infections presented by pregnant women, and the lack of resources in LMICs must also be taken into account. Consequently, continuous surveillance of the virulence and antimicrobial resistance patterns of these isolates is needed, especially in areas with adverse socio-economic conditions.
